# An excess vessel in the posterior part of the human cerebral arterial circle (CAC): a case series

**DOI:** 10.1186/1471-2377-10-53

**Published:** 2010-06-23

**Authors:** Ljiljana Vasović, Milena Trandafilović, Ivan Jovanović, Aleksandra Antović, Jovan Stojanović, Miodrag Zdravković, Miroslav Milić

**Affiliations:** 1 Department of Anatomy, Faculty of Medicine, Blvd. Dr Zoran Đinđić 81, 18000 Niš, Serbia; 2 Faculty of Medicine, Blvd. Dr Zoran Đinđić 81, 18000 Niš, Serbia; 3 Institute of Forensic Medicine, Faculty of Medicine, Blvd. Dr Zoran Đinđić 81, 18000 Niš, Serbia

## Abstract

**Background:**

As a continuation of the previous findings in human fetuses, accidental finding of an accessory vascular component in the posterior part of CAC of human adult cadavers inspired the authors to present and compare its posterior part configuration.

**Case presentation:**

Examination was carried out on brains of 48 human adult cadavers, routinely dissected at the Institute of Forensic Medicine. The aberrant vessel in the posterior part of four CACs was discovered.

Vascular components of the posterior segment of CAC or of the whole CAC were described and photographed. A comparison between fetal and adult cases was also presented.

**Conclusions:**

Based on the fact that the age of the four presented cases ranged from 73 to 84 and based on the causes of their death, we concluded that the angioarchitecture of the posterior part of the CAC is a consequence of the embryonic or primitive arterial stabilization and interaction with normal adult vessels.

## Background

Nine vascular components (the left and the right internal carotid artery - cerebral segment, communicating and choroid parts or ICA - C4*; pre-communicating segment of the left and right anterior cerebral arteries or ACAs-A1 or A1s; anterior communicating artery or ACoA; left and right posterior communicating artery or PCoA; and pre-communicating segment of the left and right posterior cerebral arteries or PCAs-P1 or P1 s make the cerebral arterial circle or the circle of Willis. Among them, PCoA and P1 form its posterior bilateral segments [1].

The literature information about the anomalies of diameters of PCoA and PCA cannot be disputed [1-13]. However, the insight into the angioarchitecture of the CAC posterior segment in a case of excess vascular component persistence is presented only in the Yasargil's scheme [14] and previously described fetal cases [9].

This fact inspired the author and her colleagues to add four new cases of CAC, with an accessory vascular component discovered accidentally in the human adult cadavers, to the reference database of the morphology of CAC, especially of its posterior segment.

## Case Presentation

Examination was carried out on the brains of human adult cadavers [15], routinely dissected at the Institute of Forensic Medicine of the Medical Faculty in Niš, during the period between 2006 and 2008, in accordance to the rules of the internal Ethical Committee (No. 01-206-1). The brains used in autopsies originated from 48 cadavers of both sexes and of different ages (from 20 to 87) and different causes of death.

**Figure 1:**
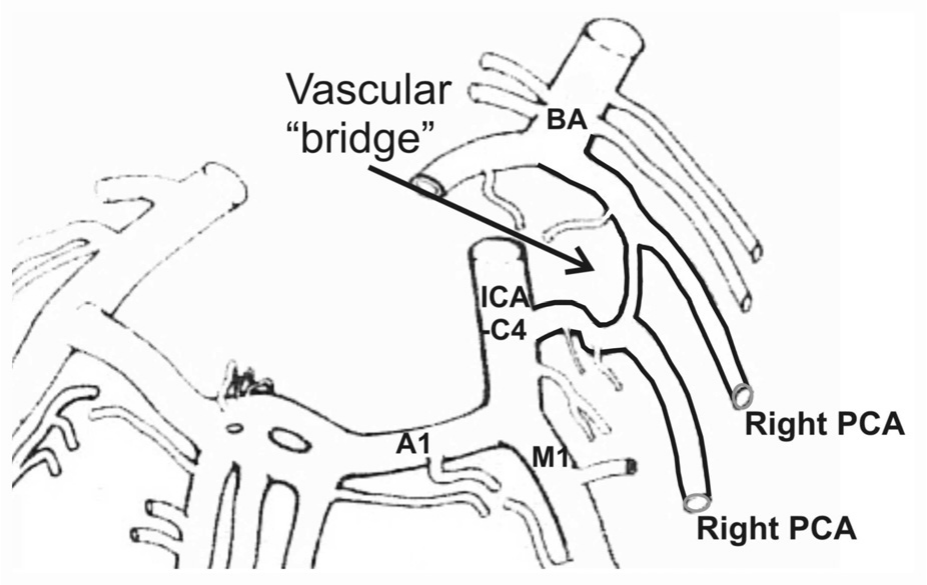
**Modified scheme **[14]** of the presence of vascular "bridge" in the posterior part of cerebral arterial circle (CAC)**. Some vascular components are named: PCA, two posterior cerebral arteries; BA, basilar artery; ICA-C4, internal carotid artery, cerebral part; A1, anterior cerebral artery, pre-communicating part; M1, middle cerebral artery, sphenoid part.

The brain base with blood vessels was photographed on each cadaver; the ventral side of the brainstem and diencephalon was specially zoomed. Each case was recorded as the schematic drawing in the workbook. External morphology (calibre of the arteries, possible abnormalities) was inspected with a magnifying glass and recorded on a film. A persistence of an excess vessel in the CAC (figure 1) is noted according to the scheme by Yasargil [14]. According to the paper by Lavieille et al [2], the anterior subpart of the artery between the ICA-C4* and an "excess" vessel is marked as the P1 segment of carotid origin (PCA-P1c), while the posterior subpart of the artery between the post-communicating part of PCA (P2) and the basilar artery (BA) is named the P1 segment of basilar origin (PCA-P1b). According to the paper by Vasović [9], the excess vascular component in the posterior segment of CAC is named as "intermediate communicating artery" (ICoA). Measurement of the outer diameter of the PCA-P1c, PCA-P1b and ICoA in two CACs was performed with ImageJ http://rsb.info.nih.gov/ij/index.html.

In the first part of this paper, individual vascular components of the posterior segment of CAC were described in four cases or the whole CAC where possible.

### Case 1 (S - 325/06)

**Figure 2:**
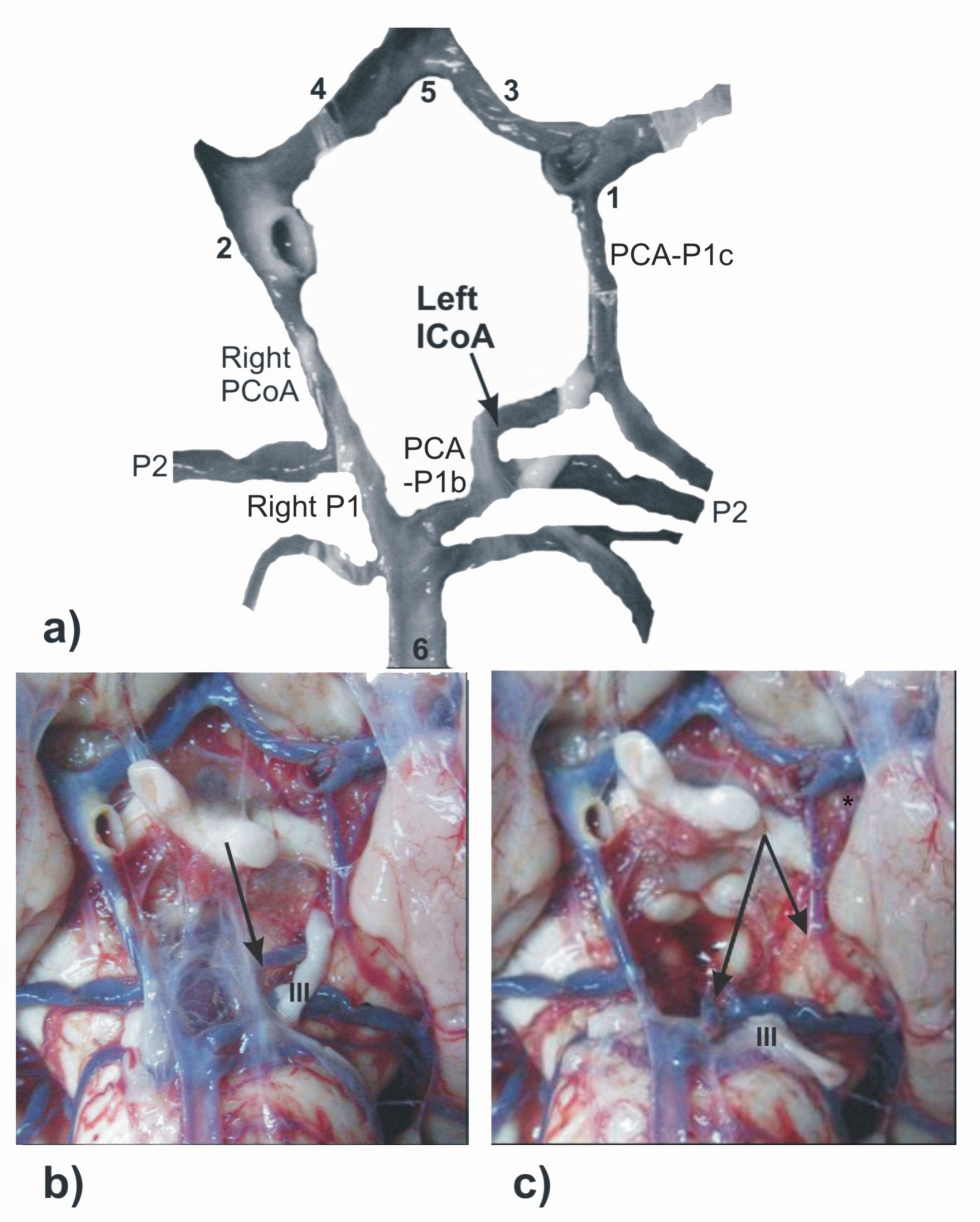
**Modified picture (a) of some cerebral arteries on the basal surface of the brain (b-c): intermediate communicating artery (ICoA) is presented on the left side in the posterior part of the CAC**. Insert **- a**. PCA-P1c, left posterior cerebral artery, pre-communicating part of carotid origin; PCA-P1b, left posterior cerebral artery, pre-communicating part of basilar origin; P2, left and right posterior cerebral arteries, post-communicating parts; PCoA, right posterior communicating artery. Some vascular components are labeled by numerals: 1-2, left and right internal carotid arteries, choroidal and communicating subparts of cerebral part; 3-4, left and right anterior cerebral arteries, pre-communicating parts; 5, anterior communicating artery; 6, basilar artery. Inserts **- b, c**. Original pictures of the cerebral angioarchitecture before (b) and after (c) move of the oculomotor nerve (III) from the ICoA (arrows). Insert **- c**. *, left anterior choroidal artery.

In the first case (a male cadaver, age 73, autopsy performed under the suspicion of intoxication), left ICoA connected parts of ipsilateral double PCAs: PCA-P1c and PCA-P1b. The ICoA had approximately the same diameter as the left PCA-P1c and right P1. The calibre of the right PCoA was larger in relation to ipsilateral P1, but equal to the calibre of the left PCA-P1b segment. The calibre of the vascular components of anterior part of CAC (ICA-C4* and A1) was relatively larger on the right side; ACoA had the same calibre as the right A1 and it was without any abnormalities. The atherosclerotic plaques were present at the wall of the ICA-C4 and intermediate part of the PCoA on the right side (figure 2).

### Case 2 (S-042/07)

**Figure 3:**
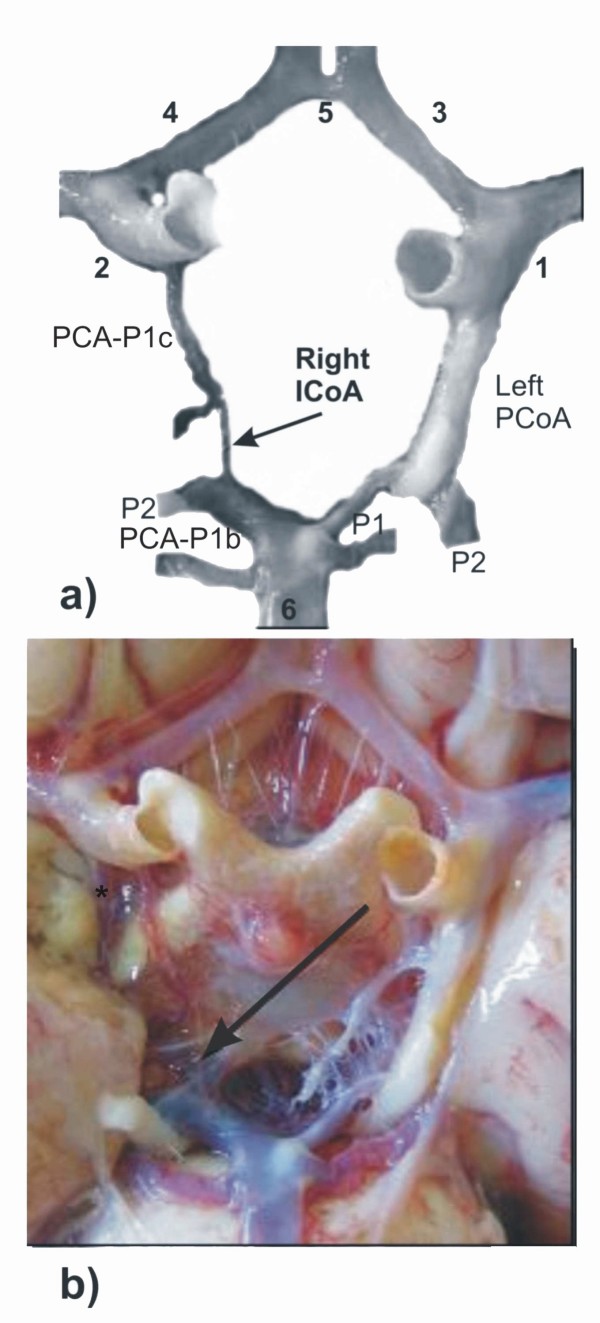
**Modified (a) and original (b) pictures of some cerebral arteries on the basal surface of the brain: hypoplastic right ICoA (arrow) is presented in the posterior part of CAC**. **Insert - a**. PCA-P1c, right posterior cerebral artery, pre-communicating part of carotid origin; PCA-P1b, right posterior cerebral artery, pre-communicating part of basilar origin; P2, left and right posterior cerebral arteries, post-communicating parts; PCoA, left posterior communicating artery. Some vascular components are labeled by numerals: 1-2, left and right internal carotid arteries, choroidal and communicating subparts of cerebral part; 3-4, left and right anterior cerebral arteries, pre-communicating parts; 5, anterior communicating artery; 6, basilar artery. **Insert - b**. *, right anterior choroidal artery.

In the second case (male cadaver, age 84, autopsy performed subsequent to his death in hospital because of cardiac arrest) the accessory artery - ICoA, of filiform appearance, was situated on the right side and it connected parts of the ipsilateral double PCAs: hypoplastic PCA-P1c and hyperplastic PCA-P1b. The calibre of the right PCA-P1c, although it was hypoplastic, was greater than the calibre of ICoA. On the left side, P1 had a slightly hypoplastic calibre, but it was greater in comparison to the calibre of the right hypoplastic PCA-P1c. The left PCoA had atheromatous plaques extending along almost the whole vessel. Its hyperplastic calibre was equalized with the calibre of the right PCA-P1b. Vascular components of the anterior segment of CAC did not show any discrepancies compared to the normal calibre. Atheromatous plaques were also present on the carotid and basilar bifurcations (figure 3).

### Case 3 (S-359/07)

**Figure 4:**
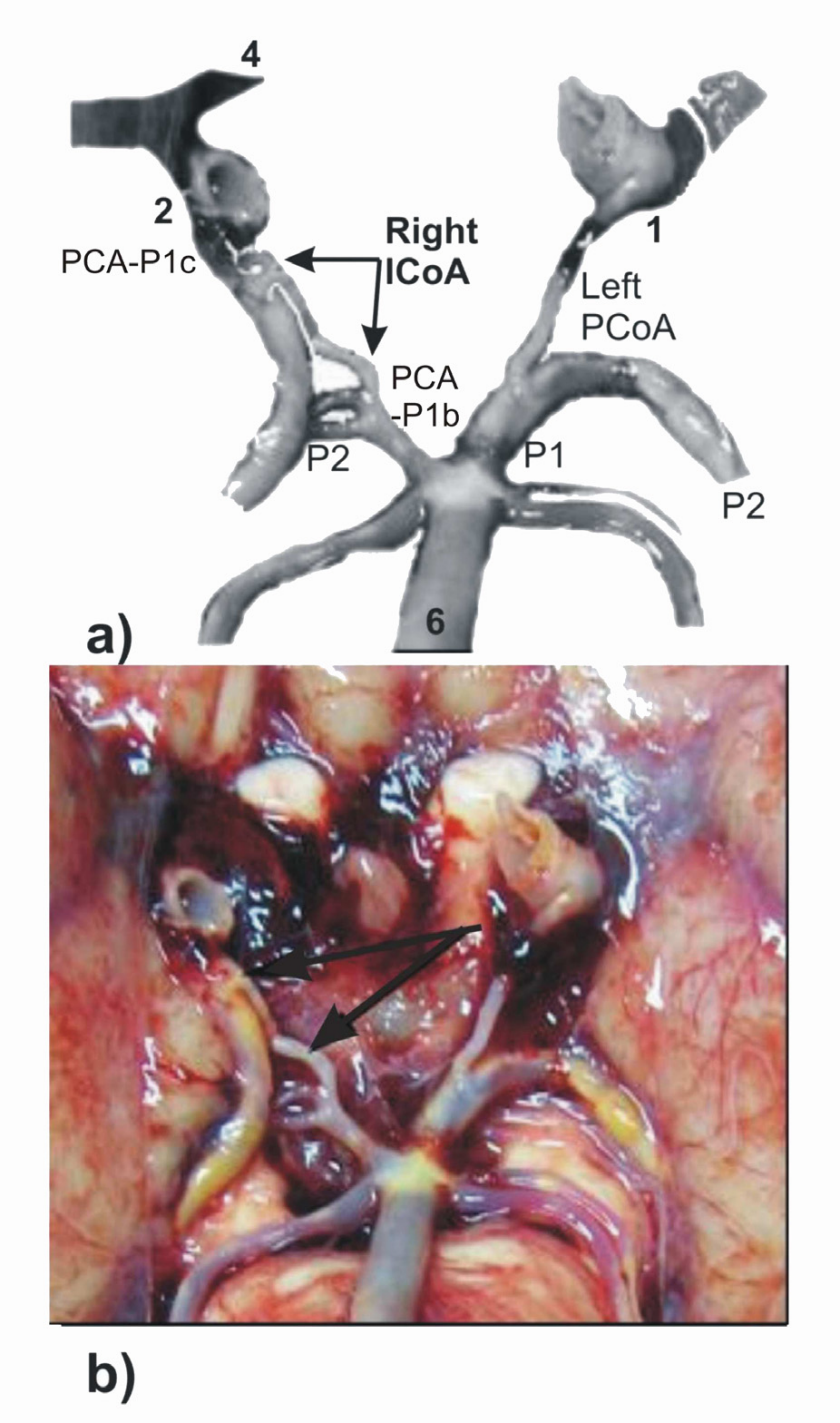
**Modified (a) and original (b) pictures of some cerebral arteries on the basal surface of the brain: right ICoA (arrows) is presented in the posterior part of the CAC**. **Insert - a**. Right posterior cerebral artery, pre-communicating part of carotid origin (PCA-P1c); right posterior cerebral artery, pre-communicating part of basilar origin (PCA-P1b); left and right posterior cerebral arteries, post-communicating parts (P2); left posterior communicating artery (PCoA). Some vascular components are labeled by numerals: 1-2, left and right internal carotid arteries, choroidal and communicating subparts of cerebral part; 4, right anterior cerebral artery, pre-communicating part; 6, basilar artery.

The third case (female cadaver, age 74, autopsy performed subsequent to severe head injury) is an example of unusual angioarchitecture of the posterior segment of CAC. The accessory artery- ICoA was situated on the right side and it connected ipsilateral double PCAs: PCA-P1c and PCA-P1b. At the level of connection with PCA-P1c, there was a bifurcation of its stem in two short branches, which converged into one unique stem - ICoA, and simultaneously caused abnormal partial duplication of PCA-P1c. The calibre of ICoA was equal to the calibre of the left PCoA. The right PCA-P1c was hyperplastic as well as contralateral P1. At the same time, the calibre of the right PCA-P1b was twice as large as the calibre of ICoA, but significantly smaller than the calibre of the left P1. The stem of the P2 segment on the right side was characterised by an early bifurcation, but we did not assess the progression and morphology of its branches. The vascular components of the anterior segment of CAC were deformed and covered with a coagulum, as well as the ipsilateral anterior choroidal artery, so that we did not examine them. Atheromatous plaques were present in the walls of the majority of the vascular components of carotid and vertebrobasilar system (figure 4).

### Case 4 (S-50/08)

**Figure 5:**
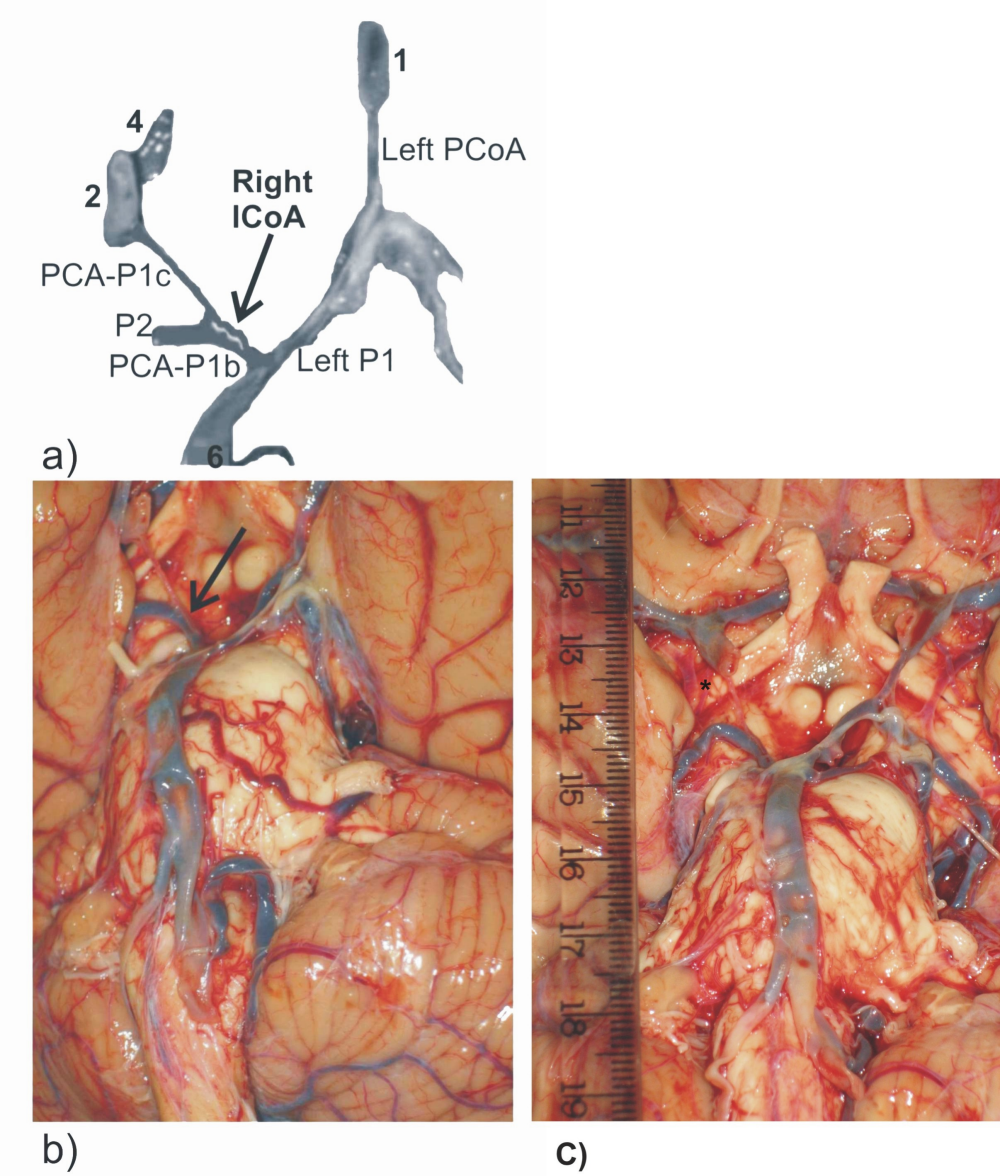
**Modified (a) and original (b-c) pictures of some cerebral arteries on the basal surface of the brain: right ICoA (arrow) is presented in the posterior part of the CAC**. **Insert - a**. Right posterior cerebral artery, pre-communicating part of carotid origin (PCA-P1c); right posterior cerebral artery, pre-communicating part of basilar origin (PCA-P1b); right posterior cerebral artery, post-communicating part (P2); left posterior communicating artery (PCoA). Some vascular components are labeled by numerals: 1-2, left and right internal carotid arteries, choroidal and communicating subparts of cerebral part; 4, right anterior cerebral artery, pre-communicating part; 6, basilar artery. **Insert - c**. *, right anterior choroidal artery.

In the fourth case (male cadaver, age 78, autopsy performed subsequent to the inflicted injury), the hypoplastic and short ICoA was situated on the right side and it connected ipsilateral double PCAs: PCA-P1c and PCA-P1b. The stem of ICoA, with almost the same calibre as that of the right PCA-P1c, passed directly along the adventitia of the same PCA-P1b segment, caused its partial duplication and connected itself with the basilar bifurcation. The right PCA-P1c had smaller calibre in comparison to the left PCoA and P1 segment. The calibre of the left P1 segment was almost equal to the calibre of the opposite PCA-P1b segment, but greater than the calibre of the left PCoA. The right ICA-C4 had greater calibre then the left one, whereas A1 segments had approximately equal calibre. Macroscopically, atherosclerotic changes were not observed in these arteries (figure 5).

In the second part of the paper we emphasized the following:

i) Table 1 presents simplified relationship of vessels' outer diameters in the posterior part of the CAC.

**Table 1:** Summarized review of relation of vessels outer diameters in the posterior part of CACs

Adult cases	Relations of arteries
Case 1.	Left PCA-P1b > left ICoA ≈ left PCA-P1c ≈ right P1 < right PCoA
Case 2.	Right ICoA < right PCA-P1c < right PCA-P1b ≈ left PCoA > left P1
Case 3.	Left PCoA ≈ right ICoA < right PCA-P1b < right PCA-P1c < left P1
Case 4.	Right PCA-P1c ≈ right ICoA< right PCA-P1b ≈ left P1 > left PCoA

ii) Figure 6 and Table 2 present the results of the CAC posterior part configuration, as well as, its components (ICoA, PCA - P1b and PCA - P1c) vascular diameters, comparing the fetal one and two selected adult cases.

**Table 2:** External diameter values (mm) of the right PCA-P1b, ICoA and PCA-P1c in one fetus and two adult cadavers

Case (sex, ageing)	PCA-P1b	ICoA	PCA-P1c
Fetus (Female, gestational weeks 23)*	0.25	0.25	0.70
Adult cadaver (Female, aged 74)	1.34	1.04	2.23
Adult cadaver (Male, aged 78)	1.34	0.56	0.82

*Ageing and diameter values of fetal arteries according to the paper of Vasović [9].

**Figure 6:**
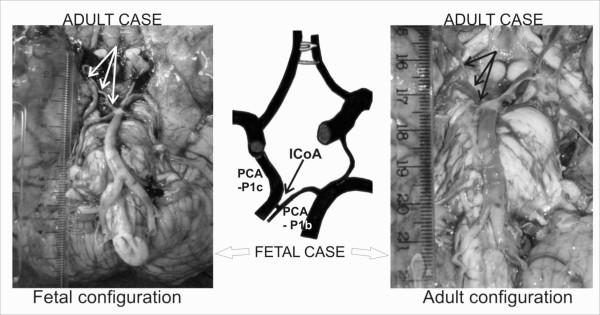
**Comparison of external diameters of the pre-communicating part of carotid origin (PCA-P1c) and pre-communicating part of basilar origin (PCA-P1b) of the right posterior cerebral arteries, as well as the right ICoA between the one fetal and two adult cases**.

This paper presents a special configuration of the posterior part of CAC with five vascular components on the brains of adult cadavers.

A generally accepted fact is that the posterior segment of CAC is normal as much as it is formed with PCoA and P1 segment on both sides, where the left and right P1 have normal calibres which are larger in relation to PCoA, as well as that PCoAs are not hypoplastic [1]. In fetuses, only one in 7 cases had normal configuration of the posterior part of CAC [9], whereas there was no such CAC in our adult cases. Independent from the existence of adult configuration in one case, the right PCoA was of a hypoplastic calibre.

We also underline the second opinion in the literature which emphasises that embryology of the posterior part of CAC is complex and that the stadiums of its development are different. We distinguish Lavieille et al [2], among other authors, who noted the descriptions by old embryologists according to whom the cranial branch of the primitive ICA in a human embryo at 20 mm of CRL incorporates to the anterior cerebral, middle cerebral and anterior choroidal arteries. Caudal branch, as the second branch of the primitive ICA, persists in the form of plexus prior to being incorporated into PCoA and PCA. There is a possibility of the caudal branch of primitive ICA simplification to the ICoA and as such vessel persists, not only in fetuses [9], but also postnatally, and this fact was supported by the four presented cases. Plexiformity of the caudal branch of primitive ICA was especially noticeable in one (third) adult case, as well as in the previous examination of the one fetus [9].

Marinković et al [7] described that PCA has a transitory origin and that PCoA is not completely developed although the embryo reached the length of 50 mm. Lie [16] described anastomoses which were formed between the caudal branch of ICA and corresponding longitudinal neural artery on the basal side of the posterior brain, in embryos of 5-6 mm crown-rump length (CRL), as well as cranial communications which were developed within PCoA.

Two ambiguous literature interpretations of cadaveric CACs should also be mentioned. Namely, although Belenkaia [3] showed similar anastomotic vessels in the left half of the posterior part of CAC, she did not mark them. Yasargil [14] only listed papers by various authors according to whom "two main branches" - PCA and PCoA, originate separately from ICA and BA, which are connected with separate branches, but without providing any figure or scheme to support this statement. Bogdanović et al [5] discovered several independent branches of PCoA which were separated from the proximal part and stretched to P1 segment, without any photo documentations. Milenković [17] described an "anastomotic loop" between the left P1 and ipsilateral PCoA using 21-week-old fetal brain, having the same ICoA localization. The latter cited author told us that he was "surprised" by the presence of such a vessel which had not been previously described in the literature, so that he only marked it as an anastomotic loop and did not discuss it further. Contrary to that, in Yasargil's book [14] we can find an analogue blood vessel in the scheme of CAC, presented in the form of the right "bridge", but without any accompanying text.

Having in mind this obvious disagreement of the data about the origin of P1 and PCoA in the relevant literature, the author felt free to suggest and describe three persistent forms of cranial extension of the primitive ICA: 1) peribasilar vessel, 2) partial or total abnormal duplication of PCoA and P1 segment, and 3) ICoA [8,9].

Unilateral persistence of ICoA, as an important characteristic of the primitive carotid-vertebrobasilar anastomoses in fetal or adult period [16], is one more reason to consider ICoA as its possible member. However, it was interesting to emphasize that, although there were a considerable number of CAC cases in human fetuses, Milenković et al [4] and Van Overbeeke et al [6] did not describe any case of ICoA. Tanaka et al [13] wrote a prospective study of CAC variations within 125 healthy volunteers, also without any cases of ICoA.

It is a fact that the anterior choroidal artery (AChA) can be confused with the PCA-P1c. Baskaya et al [12] reviewed surgical and angiographic anatomy of the PCoA and AChA. They only noted that double or even multiple AChA had been reported in 30% of the literature cases. However, they did not also describe any possibility of the ICoA presence. Despite the small diameter of AChA, usually less than 1.0 mm, we were able to identify the artery in 3 out of 4 cases simultaneously with the presence of PCA-P1c. Anatomic features helping us to identify AChA are its typical point of origin from the posterior wall of the ICA distal to the PCoA and proximal to the intracerebral carotid bifurcation, and its characteristic posterior course [18].

In the previous paper [9], two possibilities of ICoA persistence were stated. The first possibility related to coincidence, and the second one to the "neglected finding" due to the ways of measuring the calibre of P1 and PCoA by the latter as well as other authors. After this article, we believe that the second explanation could be the right one. As a confirmation of this hypothesis, we emphasise the third case (figure 3). It is obvious that if we wanted to identify ICoA with the P1 segment, a question arises considering the proper calibre of P1, whether it is the calibre of ICoA or the calibre of P1 before bifurcation.

Hypothetically, CAC posterior part fetal configuration (fetal case), could postnatally retain its status, as in the adult case 3, or it could "evolve" into adult configuration, like in the adult case 4 (figure 6). There is a possibility of its transitory pattern, when the PCoA and P1 would be equal, but such an adult case did not exist. The presence of hypoplastic ICoA in a closed fetal CAC was more frequent on the left side [9], whereas in adults, its persistence was relatively more frequent on the left side (6 out of 7 fetal and 1 out of 4 adult CACs). Since we are talking about a small series of cases, future studies are warranted to investigate the histological and morphological comparisons of PCoA-ICoA-PCA complex, as well as the comparison of their perforators.

Van Overbeeke et al [6] concluded that the variations of the posterior part of CAC were the result of the occipital lobe development. While taking into account potential confounding factors, Efekhtar et al [19] only concluded that based on many studies, there was no evidence suggesting that the distributions of the variations of CAC differ in different populations.

Hypotheses by previous authors [8,9], which cited that arteries maintain their relationship due to constant interaction between persistent primitive arterial remnants and adult cerebral arteries, might indirectly point to the conclusion that these arterial remnants can be used for ICoA formation within this rare form of CAC in fetuses. The results of our research described in this manuscript support the hypothesis that such remnants could also be responsible for ICoA persistence in postnatal period.

## Conclusions

Having in mind the fact that the reported cases are the cadavers aged 73 to 84 and the absence of cerebrovascular pathology as a direct cause of death, one could perhaps draw a conclusion that variable angioarchitecture of the posterior part of CAC represented the consequence of ICoA presence, which was in constant interaction with normal adult vessels through stabilization of its vessel configuration.

## Abbreviations

(CAC): Cerebral arterial circle; (ICA - C4*): Internal carotid artery - cerebral segment, communicating and choroid parts; (ICA - C4): Internal carotid artery - cerebral segment; (ICA): Internal carotid artery; (ACA-A1 or A1): Pre-communicating segment of the anterior cerebral artery; (ACoA): Anterior communicating artery; (PCoA): Posterior communicating artery; (PCA-P1 or P1): Pre-communicating segment of the posterior cerebral artery; (PCA): Posterior cerebral artery (PCA-P1c): P1 segment of carotid origin; (PCA-P1b): P1 segment of basilar origin; (P2): Post-communicating part of PCA; (ICoA): Intermediate communicating artery; (BA): Basilar artery; (AChA): Anterior choroidal artery;

## Competing interests

The authors declare that they have no competing interests.

## Authors' contributions

LV, MT and IJ carried out the data extraction, performed the analysis and drafted the manuscript. AA, JS, MZ and MM are individually made four forensic dissections. The figures were performed by LV and MT. All authors read and approved the final manuscript.

## Pre-publication history

The pre-publication history for this paper can be accessed here:

http://www.biomedcentral.com/1471-2377/10/53/prepub
